# Health and Advertised Remedies

**Published:** 1919-03-08

**Authors:** 


### HEALTH AND ADVERTISED REMEDIES.

With the task of improving the health of our so-called C. 3 population, the authorities, both medical and civil, have so much on their hands that many a small factor in decline may be quite easily missed. One of these, by no means the least insidious, arises from the sale of the home-remedy and the methods of its advertisement.

The proprietor of a patent medicine with his trade-mark monopoly naturally finds extensive advertising and a certain secrecy in composition extremely profitable when catering for people of limited education. One does not in any sense refer to the many excellent preparations supplied to the profession and public alike and most tactfully described in a manner understandable by both, but rather to the occasional gross abuse of advertisement in the general press, making the patent medicine business in many instances a menace to public health. The essential difference between these types lies in the fact that whereas the former, chiefly if not exclusively, notifies the public where and how its demands may be supplied, the latter is based largely upon the principle of creating demands for the products advertised. In the case of ordinary merchandise there is every excuse for attempts to form in the minds of the public a desire for things it would not otherwise want, for the advisability of such procedure with a drug preparation is more than doubtful.

No one is justified in advertising in a manner liable to make fit persons think they are ailing or to make the weak imagine they are diseased. By the skilful use of testimonials, by plausible claims, direct or inferential, the public may be led to magnify every trivial ailment and, although they may, to the manufacturers' advantage, come to consider his preparation a panacea for all disease, yet they fall into the greatest danger of overlooking the insidious onset of some really malignant illness. Many conditions which figure in their list of cures are far too serious to be self-treated and if left for even a short period without experienced advice can cause permanent damage to the system.

Theoretically the National Health Insurance legislation should have done much to reduce the amount of self-doctoring practised, but it is a certain fact that innumerable families are still indescriminately dosed with these "cure all" medicines, fostering both the neurotic temperament and a chronically lowered bodily tone. The responsibility does not rest with the drugs themselves—mostly they are quite harmless—but with the ruthless method of their advertisement in the lay press, and this one cannot too strongly condemn.

